# Engineering a New Polymeric Heart Valve Using 3D Printing—TRISKELION

**DOI:** 10.3390/medicina58111695

**Published:** 2022-11-21

**Authors:** Philip Tschorn, Filip Schröter, Martin Hartrumpf, Ralf-Uwe Kühnel, Roya Ostovar, Johannes M. Albes

**Affiliations:** 1 Department of Cardiovascular Surgery, Heart Center Brandenburg, Brandenburg Medical School Theodor Fontane, 16321 Bernau bei Berlin, Germany; 2 Faculty of Health Sciences Brandenburg, 14770 Brandenburg an der Havel, Germany

**Keywords:** polymeric valve, mechanical valve, biological valve, 3D printing, heart valve engineering

## Abstract

*Background and Objectives:* Developing a prosthetic heart valve that combines the advantageous hemodynamic properties of its biological counterpart with the longevity of mechanical prostheses has been a major challenge for heart valve development. Anatomically inspired artificial polymeric heart valves have the potential to combine these beneficial properties, and innovations in 3D printing have given us the opportunity to rapidly test silicone prototypes of new designs to further the understanding of biophysical properties of artificial heart valves. TRISKELION is a promising prototype that we have developed, tested, and further improved in our institution. Materials and *Methods:* STL files of our prototypes were designed with FreeCad 0.19.2 and 3D printed with an Agilista 3200W (Keyence, Osaka, Japan) using silicones of Shore hardness 35 or 65. Depending on the valve type, the support structures were printed in AR-M2 plastics. The prototypes were then tested using a hemodynamic pulse duplicator (HKP 2.0) simulating an aortic valve cycle at 70 bpm with 70 mL stroke volume (cardiac output 4.9 L/min). Valve opening cycles were visualized with a high-speed camera (Phantom Miro C320). The resulting values led to further improvements of the prototype (TRISKELION) and were compared to a standard bioprosthesis (Edwards Perimount 23 mm) and a mechanical valve (Bileaflet valve, St. Jude Medical). *Results*: We improved the silicone prototype with currently used biological and mechanical valves measured in our setup as benchmarks. The regurgitation fractions were 22.26% ± 4.34% (TRISKELION) compared to 8.55% ± 0.22% (biological) and 13.23% ± 0.79% (mechanical). The mean systolic pressure gradient was 9.93 ± 3.22 mmHg (TRISKELION), 8.18 ± 0.65 mmHg (biological), and 10.15 ± 0.16 mmHg (mechanical). The cardiac output per minute was at 3.80 ± 0.21 L/min (TRISKELION), 4.46 ± 0.01 L/min (biological), and 4.21 ± 0.05 L/min (mechanical). *Conclusions:* The development of a heart valve with a central structure proves to be a promising concept. It offers another principle to address the problem of longevity in currently used heart valves. Using 3D printing to develop new prototypes provides a fast, effective, and accurate way to deepen understanding of its physical properties and requirements. This opens the door for translating and combining results into modern prototypes using highly biocompatible polymers, internal structures, and advanced valve layouts.

## 1. Introduction

The biophysical and hemostaseological characteristics of artificial heart valves are decisive in selecting the most suitable prosthesis for each patient. Mechanical valves, while offering superior longevity [1], require lifelong anticoagulation. Biological valves made of flexible materials such as porcine valves or bovine pericardium are much more biocompatible and eliminate the need for anticoagulation, but have a limited life span due to calcification and valve failure [2]. An ideal valve prosthesis would combine the advantages of both types, but seven decades of research have failed to develop such a valve. One approach to achieve this is polymeric valves made of flexible but synthetic materials, such as the Gott–Daggett valve (1963) [3]. Advanced surface treatment made the polycarbonate ring with a disc of Teflon fabric resistant to clotting, but calcification and stiffening of its leaflets made this valve technically obsolete as development moved on, despite some of these valves lasting for 25 years in patients [4].

### 1.1. New Developments in Polymer and Surface Treatment

Implantation of artificial heart valves dates back to the 1960s, when pioneers began implanting Bahnson, Starr–Edwards, or Harken–Soroff prostheses into the human body [4]. The fledging field of cardiac surgery quickly developed better prostheses leading to the glutaraldehyde-treated bovine pericardial or porcine aortic valves most commonly used today [5] and pyrolytic bileaflet carbon valves [6]. 

Early developments of polymeric heart valves had major setbacks due to hydrolysis, tearing, calcification, and many more problems [7]. However, recent innovations in polymers, nanomaterials, and surface modification techniques, as well as the development of new biomaterials, have revived the idea of developing a prosthetic heart valve using polymeric structures [8]. 

One such example is represented by nanocomposites of polyhedral oligomeric silsesquioxane nanoparticles and poly(carbonateurea) urethane polymer (POSS-PCU), which exhibit high oxidation, hydrolysis, and calcification resistance [9,10], as well as excellent biocompatibility and anti-thrombogenicity through self-endothelialization [11,12].

Recent work on this polymer also showed promising results in a preclinical sheep model [13]. Other groups have demonstrated the safety and efficacy of a silver-containing device in a sheep model, which could reduce the occurrence of endocarditis [14].

In conclusion, polymer-based heart valves could avoid the risk of hemorrhage due to anticoagulation, as well as the limiting calcification of porcine and pericardial bioprostheses, and reduce the incidence of paravalvular or central leak and endocarditis [8,14,15].

### 1.2. Considerations Regarding TRISKELION

Considering this progress, we developed the design idea of TRISKELION. This is a prototype that mimics the anatomy of the biological aortic valve, with three symmetrically arranged cone-shaped cusps that add the feature of a central structure to facilitate the stability and longevity of the valve while maintaining beneficial hemodynamic and biophysical properties. 

We see great potential in TRISKELION’s central structure to reduce stress concentration on the cusp membrane, especially in the commissures and at the free leaflet edges, which can lead to fatigue damage and calcification on the sensitive cusps [16]. However, the central structure narrows the orifice area to some extent. Therefore, the design was developed under the paradigm of a large effective orifice area to keep the systolic pressure gradient low. 

In this paper, we report the research and engineering process of finding improved shapes, sizes, dimensions, and proportions to optimize hemodynamics in terms of closing time, closing volume, leak volume, regurgitation, systolic pressure gradient, and cardiac output. Our goal was to optimize the existing macrostructure of our first prototype. Using 3D printing technology, we were able to establish a rapid and accurate way to test silicone prototypes such as the PIZZA (patent DE 10 2008 012) and the TIPI valve [17] in our institution.

## 2. Materials and Methods

### 2.1. Design and Construction of Prototypes

We used FreeCAD (Version 0.19.2 for Windows) to plan and design the valve prototypes. After designing the valves, we printed them using an Agilista 3200W (Keyence, Osaka, Japan) 3D printer. The Agilista 3200W is a high-resolution 3D printer based on inkjet printing technology, which has particularly high dimension accuracy and a layer height of only 15 µm. The cold printing process results in no distortion of shape or size and offers the option of using flexible silicone (Shore hardness 35 or 65) as the printing material. For certain prototypes, we combined workpieces of both grades to combine favorable properties.

### 2.2. Hemodynamic Measurements and Adaption of the Prototype 

**Figure 1:**
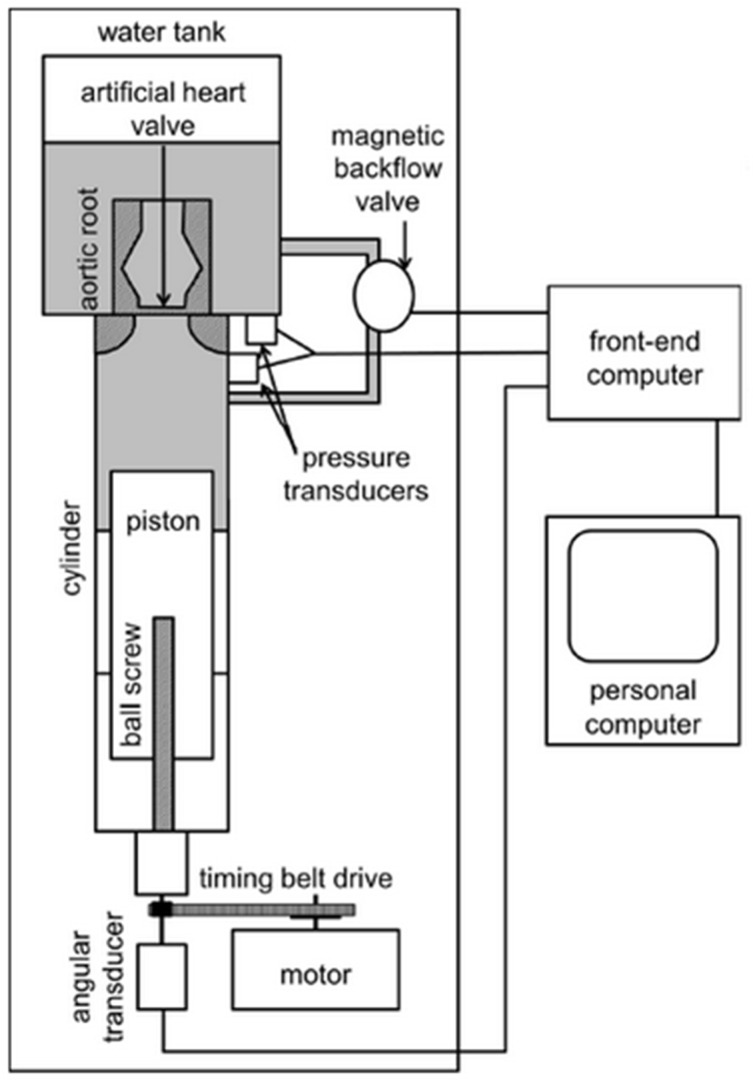
Schematic drawing of the HKP 2.0 heart valve testing device according to Schichl and Affeld.

The prototypes were tested in an HKP 2.0 hemodynamic pulse duplicator (LB engineering GbR, Berlin, Germany) according to Schichl and Affeld (Figure 1) which is an improved version of the device used, as characterized in detail in other publications [18,19,20,21].

It simulates an aortic flow profile with a heart rate of 70 bpm and a stroke volume of 70 mL, corresponding to a cardiac output of 4.9 L/min. Pressure transducers (PR 10, Keller Gesellschaft für Druckmesstechnik GmbH, Jestetten, Germany) then register the closing time and volume, leakage, regurgitation fraction, cardiac output, and mean systolic pressure gradient with an accuracy of ±1.5%. We performed at least 15 separate cycles for each valve to obtain a valid set of data.

We also visualized the test cycles with a high-speed camera (Miro C320, Phantom, Ametek, UK) for detailed observation of opening and closing characteristics and overall performance.

The results were then evaluated using Microsoft Excel 365 and led to further improvement of the TRISKELION prototype versions.

### 2.3. Data Analysis

Data analysis was performed using Microsoft Excel (Microsoft Corporation, Redmond, DC, USA) and PSPP (GNU Project). Data were tested for normality using the Kolmogorov–Smirnov test and for homoscedasticity using Levene’s tests. Normally distributed data were compared using the analysis of variance, followed by pairwise Student’s *t*-tests or Welch’s *t*-tests if variances were heterogeneous. Alternatively, the Kruskal–Wallis test, followed by pairwise Mann–Whitney U test, was used for data that were not normally distributed. To account for multiple testing, *p*-values were corrected using the Holm–Bonferroni method.

## 3. Results

**Figure 2:**
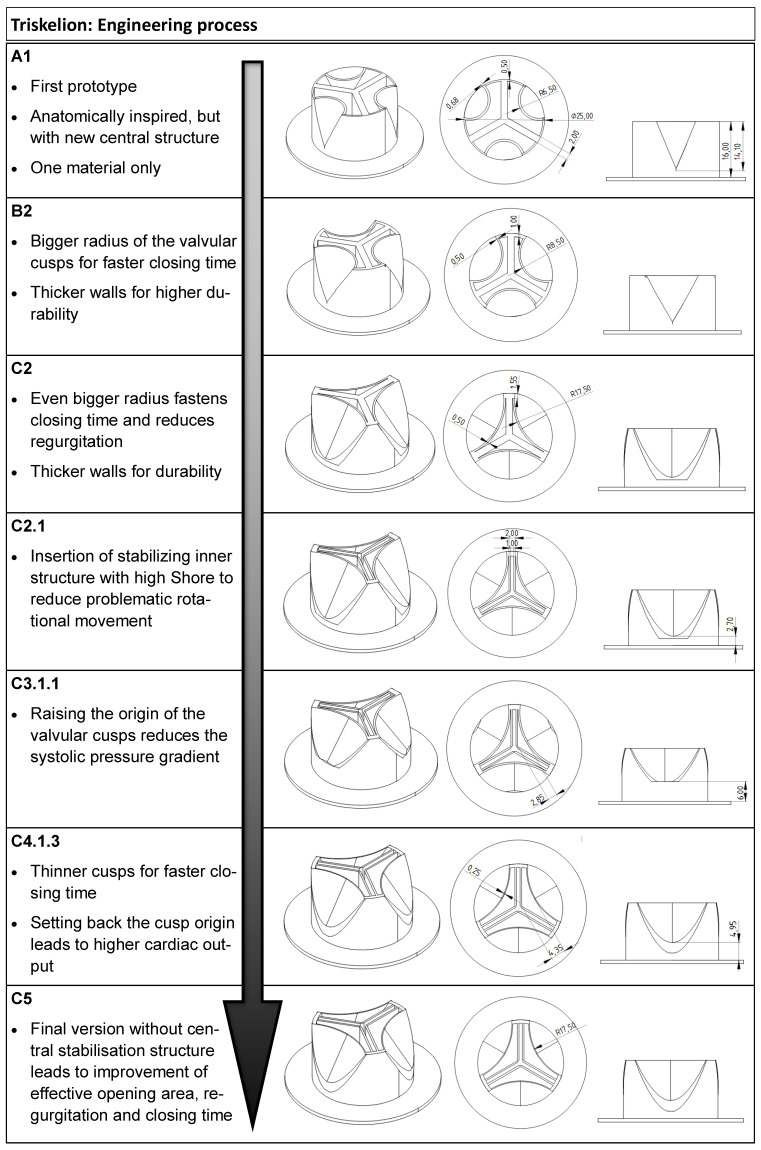
Engineering process.

**Figure 3:**
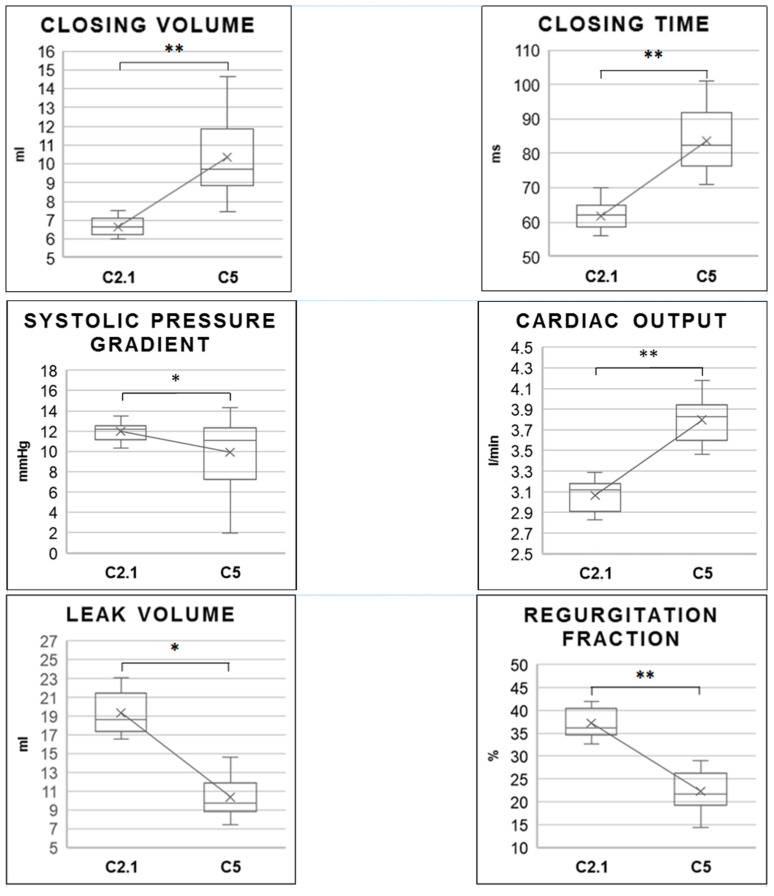
Comparison of the performance of the best-performing prototype with a solid stabilization structure (C2.1) and the last (C5) prototype with additional “inner leaflets” after removal of this structure. * *p* < 0.05; ** *p* < 0.001.

The engineering process included several steps (Figure 2). Their performance was monitored (Table 1, Figure 3) to align the development process with the goal of achieving values competitive with currently used prostheses.

***A1: Good systolic pressure gradient, but poor overall performance and stability***

Testing of the first prototype A1 revealed that overall performance needed to be improved, particularly in terms of regurgitation fraction, cardiac output, closing volume, and closing time. The first prototypes also had a regular problem of the cusps being torn apart at the base of the framework. The only outstanding parameter was a low systolic pressure gradient. 

***B2: Increasing cusp radius leads to a faster closing time***

In the next version (B2), we saw that increasing the cusp radius led to a shortening of closing time and, thus, also improved regurgitation and cardiac output without compromising the promising systolic pressure gradient. We were able to improve stability by reinforcing the stressed areas of the valves without again increasing closing time.

***C2: Closer coaptation distance, but rotational movement***

Prototype C2 was further improved by increasing the material thickness at the base of the cusps and further increasing the radius of the cusps to achieve a closer coaptation with the central structure, recognizing the side-effect of worsening the systolic pressure gradient. C2 then exhibited problematic rotational motion, especially when tested with a lower Shore material, resulting in disadvantageous stress on the cusps.

***C2.1: Reduction of the rotational movement***

Prototype C2.1 was the first version to which we added a second material with a higher Shore hardness by designing a stabilizing central structure capable of minimizing rotational movement. Thus, we were able to achieve the best values for closing volume and time to date. The goal of further research was to minimize the systolic pressure gradient, leak volume, and regurgitation fraction, and to raise the cardiac output. 

***C3.1.1 and C4.1.3: Increasing effective opening area and thinning down cusps***

We achieved a reduction in systolic pressure gradient and leak volume by raising the base of the cusps (C3.1.1), moving them apart from the central structure to obtain a larger effective opening area at the onset of systole. Thinning the cusps could further shorten the closing (C4.1.3). 

***C5: Use of the central structure as stabilizer and performance enhancer***

**Figure 4:**
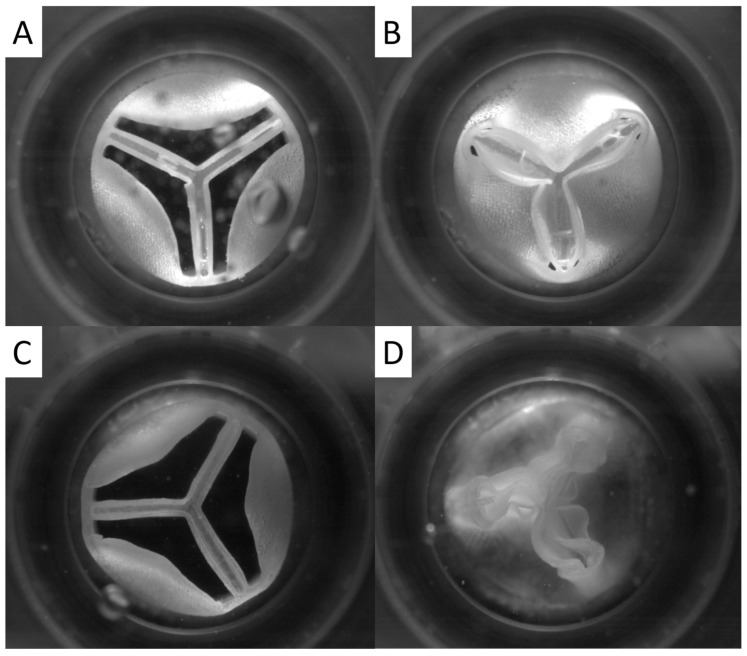
Images of prototype C5 built from silicone with Shore hardness 65 (**A**,**B**) and 35 (**C**,**D**) in open and closed states during hemodynamic testing.

In another experiment, when the higher Shore material was removed from its pocket in the stabilizing central structure (C5), we observed a valvular cusp-like movement of the remaining central structure coating toward the opposite cusps (Figure 4B,D). This resulted in a marked reduction in regurgitation fraction and leak volume, as well as a renewed increase in cardiac output (Figure 4). Although C5 had a slower closing time and larger closing volume than C2.1, the regurgitation fraction and leak volume were significantly lower. Thus, we conclude that, although this prototype had slower cusp motion, it had lower leakage, making it superior in overall performance. 

In summary, we were able to design a unique heart valve with a distinctive central structure that exhibits an excellent systolic pressure gradient and, as a single material 3D printed prototype, performs well for the performance characteristics tested. 

## 4. Discussion

The 3D design and printing process outlined above proved to be a cost-effective, flexible, and safe way to turn ideas into a measurable model. 

We started with a very simple prototype, which we improved throughout the process. In the process, we got increasingly closer to the biological shape of aortic valves and existing prostheses. What distinguishes our model is the central structure, which remains a key element throughout the development process, leading to a working prototype without the need for any solid structures as in the currently used prostheses.

Another innovation is the inner cusp originating from this inner structure, which leads to an artificial new coaptation line due to lateral movement of these inner cusps, resulting in lower regurgitation, shorter closing times, small leak volume, and low systolic pressure gradient. In this way, we were able to achieve our research goal of improving the macrostructure of our first prototype.

Compared to the Edwards Perimont bioprosthesis and Medtronic Advantage mechanical prosthesis, the current prototype already shows excellent systolic pressure gradients (Triskelion: 9.93 ± 3.22 mmHg, Perimount: 8.18 ± 0.65 mmHg, Advantage: 10.15 ± 0.16 mmHg) but still needs to be improved with respect to its regurgitation volume, which is still about twice as high as the prostheses used in comparison (Triskelion: 22.26% ± 4.34%, Perimount: 8.55% ± 0.22%, Advantage: 13.23% ± 0.79%).

Following these current results, future work will include prototypes for different annulus sizes, including 21 and 25 mm, to test the competitiveness of the demonstrated approach under more challenging hemodynamic conditions. This is particularly true for smaller annulus sizes, where the relationship between the central support structure and orifice area becomes less favorable.

While the addition of the open space in the upper third of the type C5 central structure improved overall hemodynamic performance, we are aware that this opening, like the central support structure itself, may be a source of thrombogenicity. We plan to address these issues by adding wash-out openings to reduce dead-flow areas and by testing variations in internal shape from the current sharp angles.

The silicone currently used, which we chose for its suitability for 3D printing, will not be the final material when this prototype reaches the stage of in vitro testing, as silicone has proven to be a suboptimal material for cardiac prostheses. Instead, our long-standing goal is to produce an optimized prototype using highly biocompatible polymers such as POSS-PCU. Therefore, we refrained from hemocompatibility experiments with our current prototype, as it would have been unclear whether thrombogenic events were caused by the shape or the suboptimal polymer.

**Figure 5:**
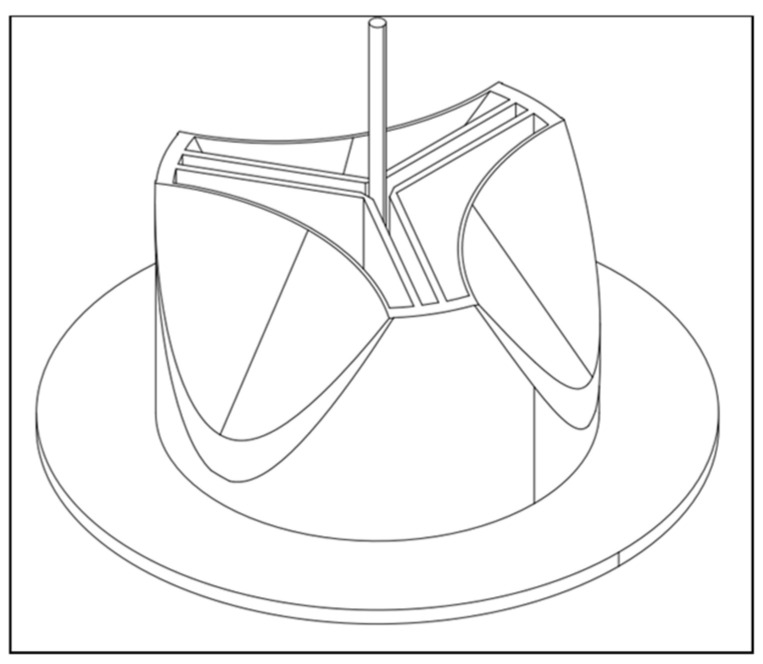
Potential insertion of TAVI guidewire through the central structure of prototype C5.

We would also like to point out that the whole prototype could be further developed to support a transcatheter implantation due to its flexibility. A small tube for the required wire could easily be placed in the center of the support structure (Figure 5).

However, our most promising results could be achieved by printing a prototype from only one material, which was not even specifically designed for in vivo applications. The future options of printing with specialized materials, surfaces, new biomaterials, or internal polymeric structures to optimize the microstructure are interesting new research projects.

Further work will also focus on options of shapes, stiffness, and size of the cusps to further optimize closing time, closing volume, and resulting regurgitation. This will also include testing other hemodynamic parameters of our prototypes. Different stroke volumes, stroke rates, and pressures are important variables to further approximate in vivo conditions.

The optimized shapes and design principles identified can then be applied to highly biocompatible polymers such as POSS-PCU to produce a competitive end product that combines the advantageous hemodynamics of biological prostheses with the longevity of mechanical prostheses. 

This will then allow us to collect advanced data on thrombogenicity and degeneration from future projects in vitro and in vivo.

## 5. Conclusions

All in all, silicone 3D printing enables rapid development and exploration of the optimal physical shape of the new TRISKELION prototype, but it cannot yet compete with existing alternatives on the valve market. Nevertheless, its low complexity, its potential cheap production, and promising developments in polymers, nanomaterials, surface technology, and inventions of new biomaterials could make it an interesting alternative.

## Figures and Tables

**Table 1:** Performance characteristics of the presented prototypes. For comparison, values are given for a biological (Edwards Perimount 23 mm) and mechanical prosthesis (Medtronic Advantage 23 mm) within our measurement setup.

	A1	B2	C2	C2.1	C3.1.1	C4.1.3	C5	Peri- Mount	Medtronic Advantage
Closing time (ms)	118.00 ± 15.94	103.67 ± 2.87	97.58 ± 8.16	61.92 ± 4.07	72.67 ± 6.07	81.73 ± 5.41	86.96 ± 13.40	42.33 ± 7.15	40.00 ± 7.03
Closing volume (mL)	20.15 ± 2.70	17.60 ± 1.22	12.96 ± 1.55	6.66 ± 0.46	7.92 ± 0.51	9.70 ± 0.63	10.37 ± 2.03	2.78 ± 0.26	2.87 ± 0.49
Leak volume (mL)	35.21 ± 1.97	31.58 ± 2.28	11.70 ± 8.50	19.34 ± 2.17	12.78 ± 6.75	10.21 ± 7.92	5.16 ± 3.35	3.18 ± 0.34	6.67 ± 0.57
Cardiac output (L/min)	1.01 ± 0.08	1.44 ± 0.21	3.15 ± 0.65	3.06 ± 0.15	3.44 ± 0.50	3.48 ± 0.59	3.80 ± 0.21	4.46 ± 0.01	4.21 ± 0.05
Regurgitation fraction (%)	79.34 ± 1.68	70.52 ± 4.42	35.36 ± 13.24	37.25 ± 3.13	29.62 ± 10.24	28.54 ± 12.11	22.26 ± 4.34	8.55 ± 0.22	13.23 ± 0.79
Systolic pressure gradient (mmHg)	8.60 ± 0.13	11.77 ± 0.50	11.17 ± 3.57	11.97 ± 0.89	9.87 ± 0.40	8.03 ± 0.53	9.93 ± 3.22	8.18 ± 0.65	10.15 ± 0.16

## Data Availability

Not applicable.
